# Development of a Finger-Ring-Shaped Hybrid Smart Stethoscope for Automatic S1 and S2 Heart Sound Identification

**DOI:** 10.3390/s21186294

**Published:** 2021-09-20

**Authors:** Soomin Lee, Qun Wei, Heejoon Park, Yuri Na, Donghwa Jeong, Hongjoon Lim

**Affiliations:** 1 Department of Biomedical Engineering, Graduate School of Medicine, Keimyung University, Daegu 42601, Korea; e_soomin@naver.com; 2 Department of Biomedical Engineering, School of Medicine, Keimyung University, Daegu 42601, Korea; hjpark@kmu.ac.kr; 3 Department of Craft Design, College of Fine Arts, Keimyung University, Daegu 42601, Korea; yurina@kmu.ac.kr; 4 Mechasolution Co., Ltd., Daegu 42715, Korea; roboholic84@gmail.com (D.J.); hongjun9110@gmail.com (H.L.)

**Keywords:** finger-ring shape, heart sounds, phonocardiogram, photoplethysmogram, Shannon entropy, stethoscope

## Abstract

Cardiac auscultation is one of the most popular diagnosis approaches to determine cardiovascular status based on listening to heart sounds with a stethoscope. However, heart sounds can be masked by visceral sounds such as organ movement and breathing, and a doctor’s level of experience can more seriously affect the accuracy of auscultation results. To improve the accuracy of auscultation, and to allow nonmedical staff to conduct cardiac auscultation anywhere and anytime, a hybrid-type personal smart stethoscope with an automatic heart sound analysis function is presented in this paper. The device was designed with a folding finger-ring shape that can be worn on the finger and placed on the chest to measure photoplethysmogram (PPG) signals and acquire the heart sound simultaneously. The measured heart sounds are detected as phonocardiogram (PCG) signals, and the boundaries of the heart sound variation and the peaks of the PPG signal are detected in preprocessing by an advanced Shannon entropy envelope. According to the relationship between PCG and PPG signals, an automatic heart sound analysis algorithm based on calculating the time interval between the first and second heart sounds (S1, S2) and the peak of the PPG was developed and implemented via the manufactured prototype device. The prototype device underwent accuracy and usability testing with 20 young adults, and the experimental results showed that the proposed smart stethoscope could satisfactorily collect the heart sounds and PPG signals. In addition, within the developed algorithm, the device was as accurate in start-points of heart sound detection as professional physiological signal-acquisition systems. Furthermore, the experimental results demonstrated that the device was able to identify S1 and S2 heart sounds automatically with high accuracy.

## 1. Introduction

According to a World Health Organization (WHO) report, cardiovascular disease is the leading cause of death worldwide, responsible for 8.9 million deaths in 2019 [1]. However, a portion of these deaths could be prevented through early diagnoses based on monitoring cardiovascular-related physiological signals such as heart sound, heart rate, electrocardiogram (ECG) readings, and blood pressure [2,3,4,5]. Therefore, a variety of approaches to detect the early symptoms of cardiovascular disease based on physiological signal measurement technologies have been developed. A Holter monitor is one of the most popular professional ECG acquisition devices, featuring a dozen electrodes that attach to the surface of the user’s chest and record ECG variation over a 24 h period. The recorded ECG data is expressed in raw data that can only be analyzed by medical staff and is thus not suitable for personal use. Health bands and smart watches with PPG sensors are also one possible solution to provide individual users with readings of their heart rates and SpO_2_ measurement via wearable technology. The small size, light weight, and strong practicality of wearable technology makes it suitable for anyone to use throughout the day. However, the displayed heart rate and SpO_2_ are calculated by sample data measured every 10 s, which introduces an error value due to incomplete data and computing processing. Furthermore, these data are measured from the wrist or fingers, not directly from the heart.

Heart sounds are the most intuitive signal for observing the status of the cardiovascular system. A normal cardiac cycle comprises four main segments: the first heart sound (S1), the systole pause interval, the second heart sound (S2), and the diastole pause interval [6]. For heart-sound auscultation, the most common method is to use analog stethoscopes with a frequency range of 20 to 200 Hz and a “bell” that is placed on the chest [7,8]. The measured sounds pass through the connecting tube to the doctor’s ears for heart auscultation.

However, ambient noise and the doctor’s level of experience affect the accuracy of the auscultation and can cause misdiagnoses. S. Mangione and L.Z. Nieman conducted a study with 453 physicians and 88 medical students to investigate the accuracy of stethoscope use in detecting heart status abnormalities [9]. All participants listened to 12 cardiac events recorded directly from patients, followed by a multichoice questionnaire, and only 20% of the cardiac events were properly recognized. With the development of digital technologies, 3M (USA) and other medical-device companies have presented various smart stethoscopes utilizing digital acoustic sensors to record the heart sound into a phonocardiogram (PCG) for signal processing and visualization [10,11,12]. However, all commercial products focus solely on signal-processing methods to analyze heart sounds, which still suffer from lower accuracy and other limitations in the diagnosis of abnormal heart status.

For a more objective method of analyzing heart sounds, multiple comparison methods for cardiovascular-system-related physiological signals have been used. R. J. Lehner et al. developed an ECG, PPG, PCG three-channel microcontroller unit (MCU) system for segmentation and characterization of the PCG signal [13]. The PCG cycle was computed along time and frequency domains using ECG and carotid pulse as references, but only the heart-murmur signal was able to be detected and classified, and the system itself was complex due to the three-channel signal measurement. Then, Shen Lu et al. demonstrated that the parameters of PPG variability (PPGV) are highly correlated with the parameters of heart-rate variability (HRV), and indicate that PPGV could be used as an alternative measurement of HRV [14]. Following this, K. Ajay Babu et al. presented a signal-processing method that can identify S1 and S2 heart sounds automatically by simultaneously recording, processing, and fusing the extracted fiducial points of the PCG and PPG signals [15,16]. The method has great potential in improving identification accuracy and robustness in the presence of both murmurs and environmental noises.

**Figure 1:**
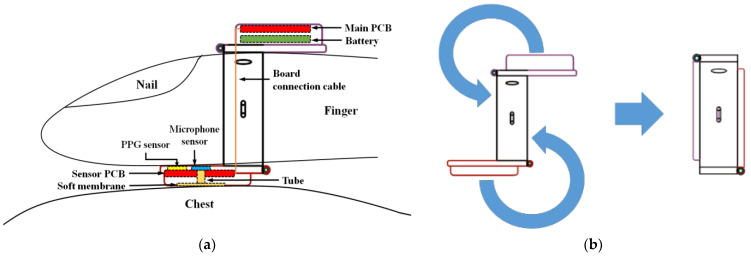
Concept of the proposed finger-ring-shaped smart stethoscope: (**a**) structure and operating principle of the smart stethoscope for PPG signal measurement and heart-sound acquisition; (**b**) collapsing mechanism of the smart stethoscope for compact portability.

Continuing from the above studies, a collapsible finger-ring-shaped hybrid smart stethoscope, as shown in Figure 1, is presented in this paper. The device can be worn on the finger and then placed on the right side of the chest where the second intercostal space and parasternal line intersect for simultaneous PPG signal measurement and heart-sound acquisition [17]. The detected PCG signal and the peak of the PPG is detected in preprocessing by an advanced Shannon entropy envelope. According to the relationship between heart sound and PPG signal, an automatic PCG analysis algorithm based on computing the time interval between S2 and the peak of PPG was developed, and implemented through a high-performance Bluetooth Low Energy (BLE) SoC ARM processor for system control, data processing, and wireless communication. The computed data was transmitted to an Android-OS-based smartphone application for data display and storage. The prototype device was manufactured and subjected to accuracy and usability testing by: comparing its readings with those of a professional physiological signal acquisition device; enlisting 20 young adults to test the performance of PPG measurements and heart-rate acquisition; and verifying the accuracy of the developed automatic heart-sound-analysis algorithm in S1 and S2 heart-sound extraction.

## 2. Methods

### 2.1. Development of the Automatic Heart-Sound-Analysis Algorithm 

The proposed hybrid smart stethoscope was not only designed for heart-sound auscultation, but also for automatic heart-sound analysis. Because one cycle of the PPG signal occurs between the first systole and the next systole, the key idea of this device is to measure heart sounds and the PPG signal simultaneously, and then, using the PPG signals in the same cycle as a reference, to identify the S1 and S2 heart sounds. Before the identifying process, there are two preprocessing phases that use the raw data of the heart sounds and the PPG signals measured by the sensors in the device: one that determines the boundaries of S1 and S2 heart sounds, and another that detects the peak of PPG signals.

**Figure 2:**
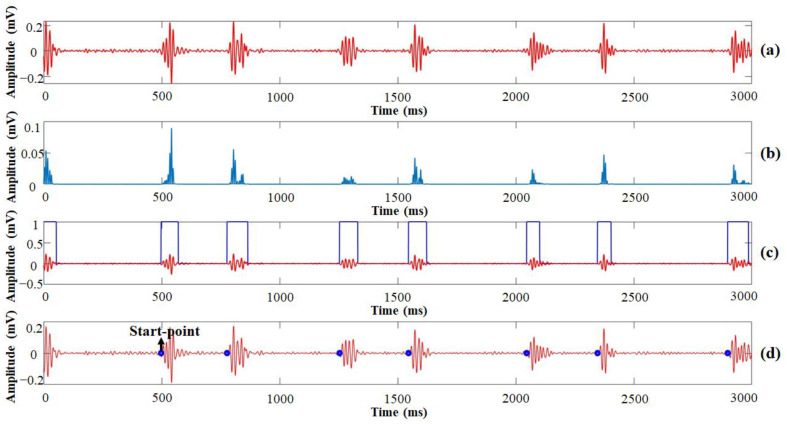
The procedure of heart sound preprocessing to determine boundaries of S1 and S2 heart sounds: (**a**) detected PCG signals; (**b**) smooth Shannon entropy envelope; (**c**) gate signal after envelope thresholding; (**d**) start-point detection.

Figure 2 shows the procedure of heart-sound preprocessing from the raw data to the boundary determinations based on variational mode decomposition (VMD) [9,18,19]. The heart sounds pass through a digital filter that rejects baseline components and high-frequency noises first, from which the PCG signal, including S1 and S2 sounds, and systolic murmurs are detected. Due to the likely existence of varying peak envelopes in the heart sounds, a third-order Shannon entropy was implemented in this study to reduce the peak variations, following the formula:

(1) $$SE=-\frac{1}{N}\overset{N}{\underset{i=1}{\sum }}\left|x_{\left(i\right)}^{\;\;\;\;3}\right|log\left|x_{\left(i\right)}^{\;\;\;\;3}\right|$$

where x is the PCG signal processed by the adaptive amplitude thresholding rule to reduce the number of false positives, and N is the length of the moving integration window, which was set to 20 samples (20 ms) with a sampling frequency of 1 kHz to be compliant with the duration of S1 and S2 heart sounds [16]. With a smoothing process based on linear zero-phase filtering with a rectangular impulse response, the multiple peaks and spikes of the PCG signals were reduced, as shown in Figure 2b. The boundaries of the local heart sounds were found by the envelope threshold rule as defined below:

(2) $$GL_{\left(i\right)}=\{\begin{array}{c} 1\;\;\;\;\;\;\;\;\;\;\;\;\;\;\;\left(0.01+SE_{avg}<SE\right)\\ 0\;\;\;\;\;\;\;\;\;\;\;\;\;\;\;\;\;\left(Otherwise\right)\;\;\;\;\;\;\;\;\;\;\;\;\;\;\;\; \end{array}$$

where $SE_{avg}$ is the mean value of the Shannon entropy (SE) as calculated by Equation (1), and GL is the gate signal. The determined boundary of the PCG signals that indicated the start-point and end-point of the heart sounds by distinguished positive and negative edges, respectively, are shown in Figure 2c. The final results of the heart-sound preprocessing are shown in Figure 2d, demonstrating that preprocessing was capable of accurately locating the start-points of the heart sounds.

**Figure 3:**
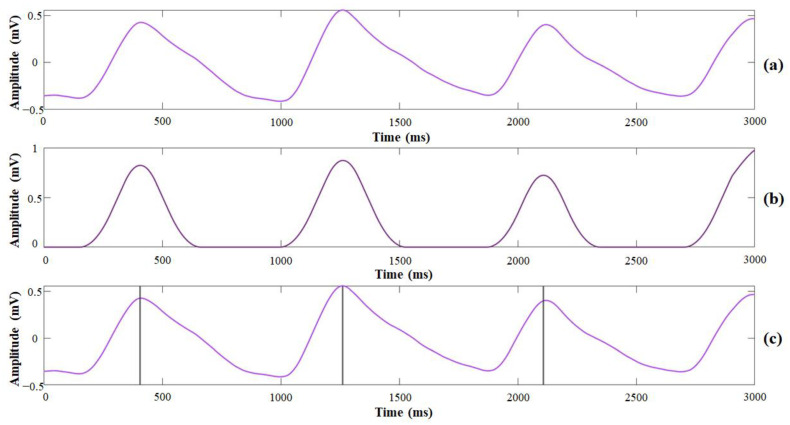
The procedure of peak point detection from the PPG signal: (**a**) recorded PPG signal; (**b**) smooth Shannon entropy envelope; (**c**) detected pulse onset and peak points.

Figure 3 shows the procedure of the PPG signals as processed from pulse waveform delineation to the systolic peak detection. To reduce background noise, the frequency threshold range was set between 0.5 and 5 Hz, which accorded with the maximum heart rate, 220 bpm [20]. A smoothing algorithm was implemented to smooth the PPG signals as follows:

(3) $$SD_{n}=\frac{\sum_{i=1}^{n}A_{i}}{n}$$

where n is the number of moving averages, A is the noise-reduced PPG signal, and the number of zero crossings is set to 10. Shannon entropy also was implemented in the PPG signal preprocessing to determine the systolic peak. The location of the systolic peak was determined by finding the maximum of the windowed sequence, which is centered at the detected peak location with a spread of 10 samples.

The S1 heart sound corresponds with the systole, and the time it takes for blood to flow from the heart to the finger is less than 500 ms. In addition, the S2 heart sound always occurs around the peak of the PPG signal ± 20 ms [21,22,23,24,25]. Therefore, an identification rule was developed to distinguish the S1 and S2 heart sounds by calculating the time interval between the peak of the PPG signal and the start-point of heart sound as follows:

(4) $$HS_{sp}=\{\begin{array}{c} S1_{sp}\;\;\;\left(T_{peak}-500\;\mathrm{ms}\leq T_{sp}<T_{peak}-20\;\mathrm{ms}\right)\;\\ S2_{sp}\;\;\;\left(T_{peak}-20\;\mathrm{ms}\leq T_{sp}\leq T_{peak\;}+20\;\mathrm{ms}\right)\;\;\; \end{array}$$

where $T_{peak}$ is the time of the PPG signal’s peak, and $T_{sp}$ is the time of the PCG signal’s start-point in the same cardiac cycle. The start-point of S1 sound ($S1_{sp}$) was distinguished in an extracted local sound segment between the $T_{peak}-500\;\mathrm{ms}$ and $T_{peak}-20$ ms in the time-domain waveform. The start-point of S2 sound ($S2_{sp}$) also was distinguished by the same method, with an extracted local sound segment classified around the peak of the PPG signals between $T_{peak}-20\;\mathrm{ms}$ and $T_{peak}+20\;\mathrm{ms}$.

### 2.2. Embedded System Design of the Proposed Finger-Ring-Shaped Hybrid Smart Stethoscope

For heart sounds and PPG signal acquisition, a digital microelectromechanical systems (MEMS) microphone and a PPG sensor were selected and incorporated in the design of the proposed finger-ring-shaped hybrid smart stethoscope. The digital microphone (ICS-43434, TDK, Tokyo, Japan) had a high SNR of 64 dBA and a wideband frequency response range of 20 to 30 MHz, suitable for recording the heart-sound frequency range of 20 to 400 Hz. In addition, the sensitivity tolerance of the sensor was ±1 dB, which enabled high-performance microphone arrays without the need for system calibration. The microphone was available as a surface-mount package and had an I^2^S interface that allows it to be connected to the MCU directly. For PPG signal measurement, an ultra-low-power, completely integrated optical pulse oximeter and heart rate sensor (MAX86140, Maxim Integrated, San Jose, CA, USA) was selected for the PPG sensor. The sensor was designed with optimized architecture for heart-rate measurement and SpO_2_ monitoring. The sensor had a low-noise signal-conditioning analog front-end with 19-bit ADC, an industry-leading ambient-light-cancellation circuit, and a picket-fence detect-and-replace function. With a standard serial peripheral interface (SPI)-compatible interface, the sensor could also be connected to the MCU directly.

Due to the proposed device’s requisite small size, long hours of use, and familiar wireless communication protocol for personal usage as a finger-ring-shaped device, a BLE-supporting ARM Cortex-M4 microcontroller (EFR32MG, SiliconLabs, San Jose, CA, USA) was chosen as the main processor for system control, data processing, and wireless communication. This chip includes a 40 MHz ARM Cortex-M4 MCU with 1024 kB flash memory, 256 kB RAM, and a rich peripheral set to easily connect the digital microphone and PPG sensor. With 19 dBm maximum output power and a receiver sensitivity of −102.7 dBm, the device provides an excellent link budget for greater range and reliable Bluetooth communications. The MCU was built with innovative low-energy techniques and fast wake-up times for extended usage. In addition, the chip was packaged as a ball grid array (BGA) package, which minimized the size of the printed circuit board (PCB). The BLE contained in the MCU was intended to provide considerably reduced power consumption and lower cost while maintaining a similar communication range, using the same 2.4 GHz radio frequencies as industrial, scientific, and medical (ISM) bands, aimed at novel applications in the medical field. In addition, mobile operating systems, including iOS and Android, natively support BLE and allow for easy connection between mobile devices and the proposed smart stethoscope.

**Figure 4:**
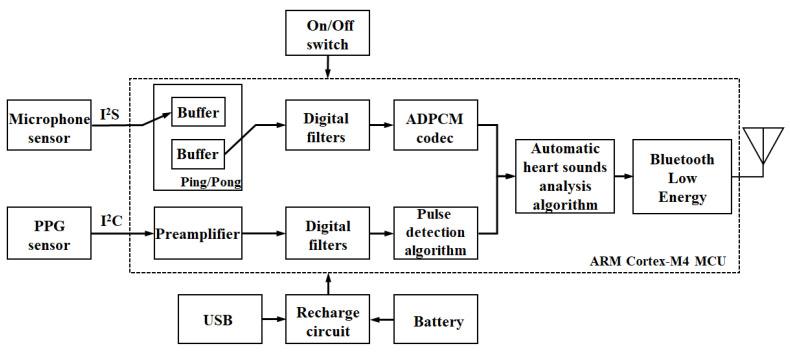
Block diagram of the proposed hybrid smart stethoscope’s embedded system.

Figure 4 shows the block diagram of the proposed smart stethoscope’s embedded system. Because the two selected sensors provided digital data output with I^2^S and I^2^C communication protocols, respectively, only heart sound and PPG signal processing had to be considered in the design of the proposed system’s digital signal processing. However, because the two physiological signals must be acquired simultaneously, the data-acquisition procedure was separated into two channels for each heart sound and PPG signal measurement, operating as parallel processes. Considering that heart sounds are an audio stream, a ping-pong buffer was designed for this system to process the heart sounds continuously, from receiving sensor data to extracting the data features. Preprocessed data of the heart sounds and PPG signals were merged and processed through the developed automatic heart-sound-analysis algorithm. Finally, the processed data was transmitted by the BLE to the smartphone application for data display and storage.

## 3. Materials and Experiment

### 3.1. Design and PCG Manufacture

**Figure 5:**
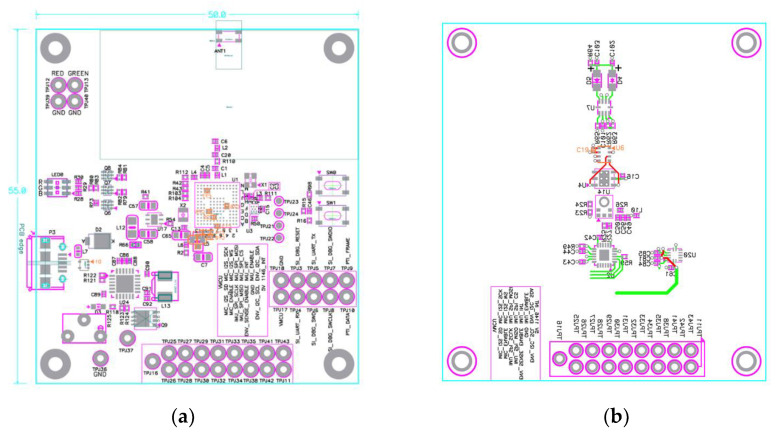
PCB artwork design of the proposed smart stethoscope: (**a**) main part; (**b**) sensor part.

To allow the proposed smart stethoscope to be folded for easy use and portability, the circuit of the device was designed and separated into two parts: the main part for de-vice control and power supply, and the sensor part for heart-sound and PPG-signal measurement. Figure 5a shows the PCB artwork of the main part, with the MCU located in the center of the board to shorten the distance from supplied electrical elements and reduce the electrical noise in the circuit. The power supply included a battery-recharge circuit and a micro-A USB port, and was designed to be on the right side of the MCU to provide a 3 V DC current to the device and to recharge the battery. A 2.4 GHz chip-shaped antenna was positioned on the top of the board to avoid electrical interference from the other components that can reduce signal strength. The sensor part’s PCB artwork design, including the acoustic sensor and the PPG sensor circuits, is shown in Figure 5b. To acquire the heart sound accurately and with maximal efficacy, the microphone was located in the center of the sensor part. The PPG sensor was fixed on the top of the layout to access the distal phalanges of the finger, which is the optimal position for measuring PPG signals. Two sets of corresponding connection pins in each part directly connected the two parts using a flat cable.

**Figure 6:**
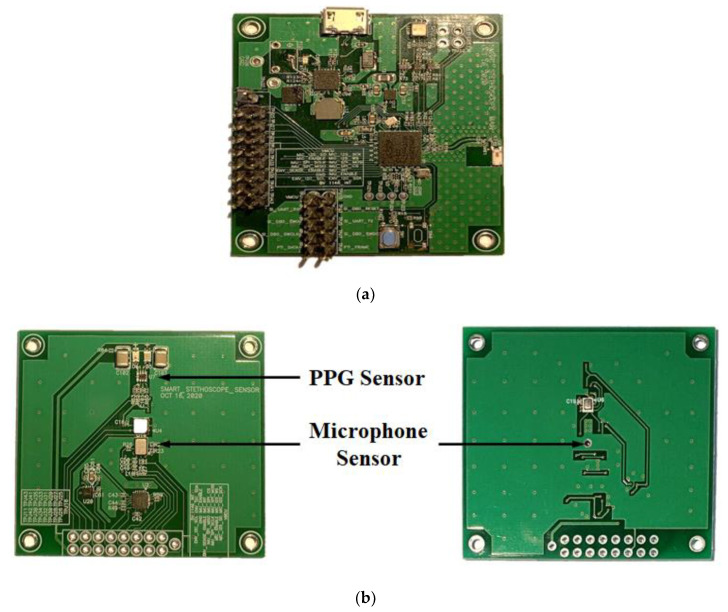
Pictures of the manufactured smart stethoscope PCB: (**a**) PCB of the main part; (**b**) PCB of the sensor part.

Following the PCB artwork design, the PCBs for prototype assembly of the proposed hybrid smart stethoscope were manufactured, as shown in Figure 6. Both PCBs were constructed as two-layer PCBs, and 1608-size electrical components were used to minimize the size of the PCBs. In addition, all components of the main board were affixed to the top of the PCB to obtain an optimized antenna pattern and reduce heat conductivity while recharging the battery. For the sensor board, because the PPG sensor must face the user’s finger, the PPG sensor was affixed to the top of the PCB with 2 LEDs for light transmission. The microphone was also attached to the top of the PCB due to the location of the sound-acquisition hole on the bottom of the microphone. Therefore, a small hole was drilled through the PCB under the sensor to connect to the membrane of the stethoscope’s head for heart-sound transmission.

### 3.2. Case Design and Prototype Assembly

**Figure 7:**
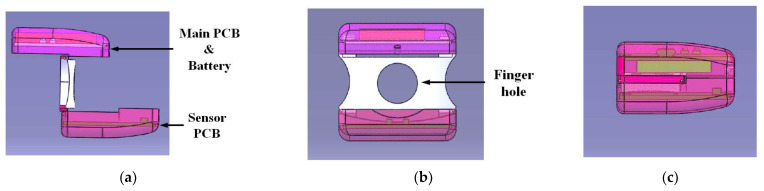
Pictures of 3D models of the proposed finger-ring-shaped smart stethoscope: (**a**) side view in user mode; (**b**) front view in user mode; (**c**) side view in collapsed mode.

Figure 7 shows 3D models of the proposed finger-ring-shaped hybrid personal smart stethoscope in user and collapsed modes. The device’s design followed the shape of the letter “Z”, with top, middle, and bottom parts. The top and bottom parts were designed to contain the main and sensor PCBs, respectively, and the middle part to house the finger hole so the user could easily keep the device on their finger. In addition, the two lateral sides of the finger hole were designed with arches to make the device more ergonomic for the adjacent fingers. Relying on two joint connections, the top and bottom parts were connected by the middle part, which pushes the top and bottom parts apart from one another when opened for user mode, and back together for the collapsed and portable mode, as shown in Figure 7c.

**Figure 8:**
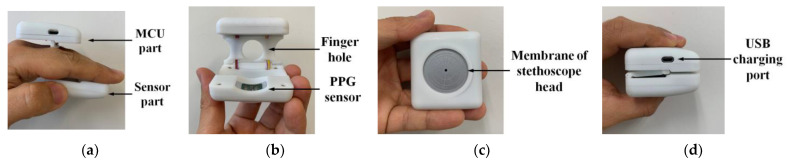
Picture of assembled finger-ring-shaped smart stethoscope prototype: (**a**) side view in user mode; (**b**) front view in user mode; (**c**) bottom view in user mode; (**d**) side view in collapsed mode.

Following the 3D model of the proposed device, its case was manufactured by a 3D printer with acrylonitrile butadiene styrene (ABS) material, as shown in Figure 8. Because the PPG sensor operation was based on an optical-measurement method, the cover of the sensor was made of transparent acrylic material, allowing light to pass between the sensor and the finger. In addition, the cover followed the contours of the finger so the finger could reach the sensor more closely. Figure 8c shows the bottom of the prototype device that was placed on the chest for heart-sound acquisition. A thinner plastic membrane, which was sensitive to heart-sound vibration was used to better capture heart sounds. A small hole with the same diameter as the hole of the microphone was also drilled in the center of the membrane for sound conduction. Sound passed from membrane to the microphone through a 3 mm-long pipe that was used to connect the sensor PCB and membrane.

### 3.3. Experiment to Test the Performance of the Proposed Smart Stethoscope

To test the performance of the hybrid smart stethoscope prototype, two goals were set in the experiments: (1) testing the accuracy of heart-sound and PPG-signal measurement; and (2) testing the accuracy of automatic S1 and S2 heart-sound detection using the developed algorithm. Twenty healthy adult subjects (gender: male; age: 24–27 years old) with no history of cardiovascular disease were invited to join this study to record the heart sounds and PPG signals. The subjects were informed of the experiment’s procedure and this study’s purpose to evaluate the smart stethoscope, and signed informed-consent forms prior to the experiments.

**Figure 9:**
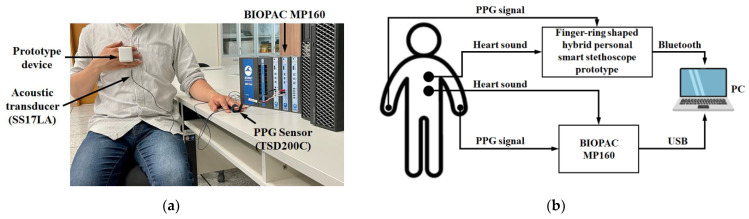
Pictures of the experimental setup for heart-sound and PPG-signal-data acquisition: (**a**) photo of a subject and measurement with the prototype device and MP160; (**b**) diagram of the experimental setup for data acquisition.

In this study, to test the performance of heart-sound and PPG-signal measurements, an MP160 multichannel physiological data-acquisition system (BIOPAC System, Inc., USA) was used as a reference for comparison with the prototype device’s data acquisition. During the experiment, the subject was asked to sit comfortably in a chair and relax for about two minutes. A contact acoustic transducer (SS17LA) and a PPG sensor (TSD200C), also provided by the MP160 system, were attached to the surface of the subject’s chest and to the middle finger of the left hand, respectively. At the same time, the subject was required to wear the prototype device on the middle finger of the right hand and to place it on the chest near the contact acoustic transducer to measure the heart sounds together. Figure 9 shows the photo of the experiment’s setup and procedure. The experiment of heart-sound and PPG-signal-data acquisition was conducted by two devices simultaneously in one minute. In addition, for data storage, a high-performance PC was used to receive and store the data from the MP160 and the prototype device by USB cable and Bluetooth, respectively.

## 4. Results and Discussion

### 4.1. Results of PCG and PPG Detection

**Figure 10:**
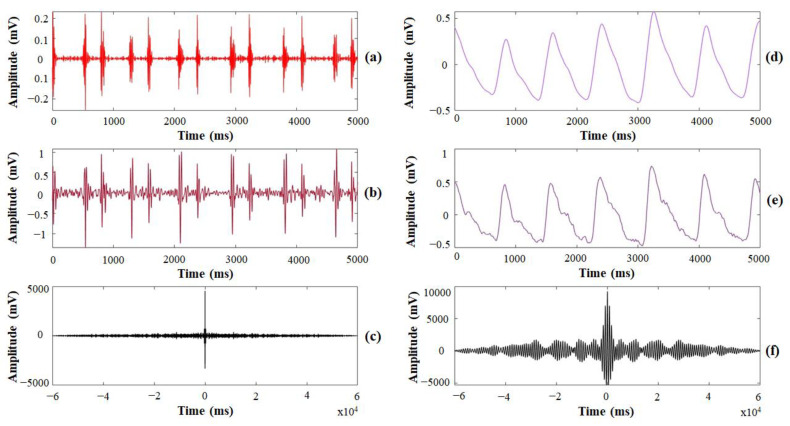
An example of the heart sounds and PPG signals measured by the prototype device and the MP160: (**a**) PCG signal detected by the prototype device; (**b**) PCG signal detected by the MP160; (**c**) cross-correlation results of the PCG signal comparison; (**d**) PPG signal detected by the prototype device; (**e**) PPG signal detected by the MP160; (**f**) cross-correlation results of the PPG signals comparison.

Figure 10 shows an example of heart sounds and PPG signals measured by both the manufactured finger-ring-shaped hybrid personal smart stethoscope prototype and the MP160 for one subject. Heart sounds measured and detected as a PCG signal by the prototype device and the MP160 are shown in Figure 10a,b. Because the PCG signal was processed with digital data directly and preprocessed to suppress environment noise and S3 and S4 heart sounds in the prototype device, S1 and S2 heart sounds were more prominent, with large signal variation, and could be distinguished better than the signals measured by the MP160. Using Pearson’s cross-correlation, similarities between the two devices’ detected PCG signals were observed to evaluate the performance of the prototype device in heart-sound measurement. As Figure 10c shows, two signals with a smaller difference in amplitude and time variation were observed.

For PPG-signal detection, the sample signals measured by the prototype device and the MP160 are shown in Figure 10d,e, respectively. Because the baseline noise and residual noise were suppressed by preprocessing after the data acquisition in the prototype device, the PPG signals acquired by the prototype device were more distinct and stable than the signals acquired by the MP160. Therefore, as Figure 10f shows, a smaller difference between two signals was observed after the two PPG signals were also treated with the same cross-correlation algorithm to evaluate the performance of the prototype device in PPG-signal measurement. Table 1 shows the calculated cross-correlation value of the heart sounds and PPG signals measured by the prototype device and of the BIOPAC system for 20 subjects, with means of 0.98 ± 0.02 and 0.96 ± 0.03, respectively. Both values were approximately 1, meaning the heart sound and PPG signal measured by the prototype device and the MP160 were similar and positively correlated. Therefore, the experimental results demonstrated that the PCG signals and PPG signals detected by the prototype were credible.

### 4.2. Automatic Identification of S1 and S2 Heart Sounds

To test the performance of the proposed smart stethoscope for automatic identification of S1 and S2 heart sounds using the developed algorithm based on PPG signal reference, the measured PCG signals were interpreted by the developed algorithm to compare with the envelogram-calculation approach commonly used to find the boundaries of S1 and S2 heart sounds.

**Figure 11:**
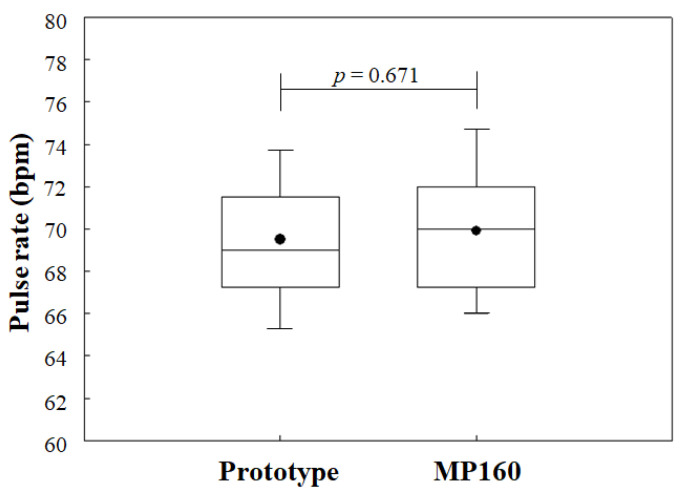
Boxplot of similarity analysis with Wilcoxon paired-samples test for heart rates of 20 subjects measured by the prototype device and the MP160.

One cardiac cycle is equal to one set of S1 and S2 heart sounds, as well as to one cycle of PPG signals. Therefore, the measured PPG signals were designated as the basis from which to calculate the heart rates of each subject and to predict the number of cardiac cycles first. As shown in Figure 11, the average heart rates of the 20 subjects measured by the prototype device and by MP160 were 68.3 ± 3.2 bpm and 69.2 ± 5.2 bpm, respectively. The Wilcoxon paired-samples test showed that two data points were similar (*p* = 0.736). Thus, the predicted total number of S1 and S2 heart sounds was set as 138 for each subject in this experiment.

**Figure 12:**
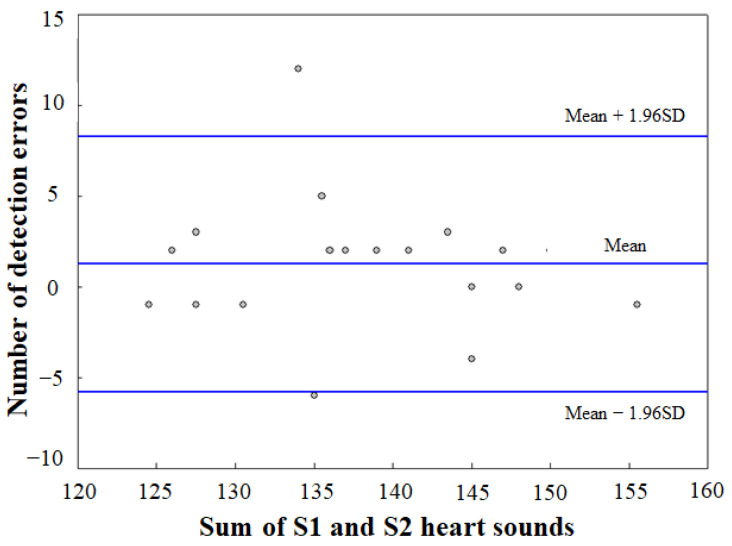
Bland-Altman plot for analysis of detected heart sounds using envelogram approach.

Based on the detected PCG signals, the total number of S1 and S2 heart sounds as calculated by the envelogram approach is shown in Figure 12. The sum totals of S1 and S2 heart sounds were 132.6 ± 5.27 as PCG signals measured by the prototype device, and 138.4 ± 8.32 as PCG signals measured by the MP160. The PCG signals measured by the prototype device were also imported as raw data into BIOPAC’s AcqKnowledge software for signal segmentation under the same conditions as the experiments, and the resulting sum totals of the prototype device were less than those of the MP160, which was assumed to be due to the resolution of the proposed device’s microphone sensor being lower than that of the MP160′s acoustic transducer, causing PCG signals to be detected with lower amplitudes. These problems were also reported in several studies for automatic identification of S1 and S2 heart sounds based on the envelogram approach with no physiological signal reference [26,27,28].

**Figure 13:**
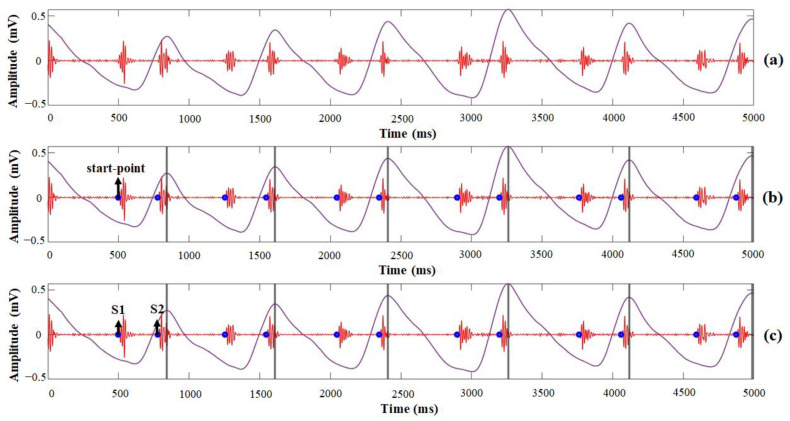
Example of procedures and results of automatic S1 and S2 heart-sound identification by the developed algorithm using a PPG signal reference: (**a**) recorded PCG and PPG; (**b**) detected start-points of heart sound and peak of PPG; (**c**) identified S1 and S2 heart sounds.

To improve the accuracy of heart-sound detection and distinguish S1 and S2 heart sounds, we attempted automatic S1 and S2 heart sound identification for one subject using the developed algorithm based on calculating the time interval between peaks of the PPG signals and start-points of the heart sounds (Figure 13). First, the detected PCG signal and PPG signal were combined in the same time domain to observe the relationship between the heart sounds and PPG signals. Second, the positive peaks of the PPG signals for each cycle were detected and the start-points of the heart sound variation were calculated simultaneously. Finally, using the identification rule as developed and mentioned in Section 2, the start-points of S1 and S2 heart sounds that referred to the time of the PPG signal’s peak were identified.

**Figure 14:**
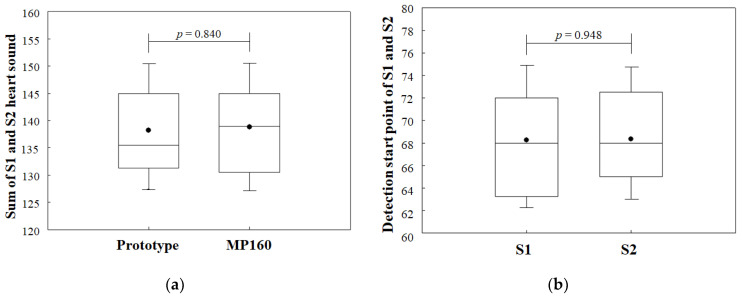
Results of the automatic identification of S1 and S2 heart sounds by the developed algorithm: (**a**) results for comparison of start-points of heart sounds detected by the prototype device using the developed algorithm and the MP160; (**b**) boxplot of the similarity analysis with a Wilcoxon paired-samples test for automatic start-points of S1 and S2 heart-sound identification using the developed algorithm.

Figure 13 shows the heart sounds interpreted by the developed algorithm to identify the start-points of S1 and S2 heart sounds. The number of start-points of heart sounds detected by the developed algorithm was 138.2 ± 7.8, which was higher than the results acquired by the envelogram approach with no PPG signal reference. In addition, the accuracy of heart-sound detection was increased to 98%, which was close to the results acquired by the MP160 with a small difference error (*p* = 0.840), as shown in Figure 14a. The performance of the developed algorithm in automatic S1 and S2 heart-sound identification, as shown in Figure 14b, with a similarity analysis carried out by the Wilcoxon paired-samples test, showed that there were no significant differences (*p* = 0.948) between the start-points of S1 and S2 heart sounds as distinguished by the developed algorithm.

The experimental results demonstrated that the proposed finger-ring-shaped hybrid personal smart stethoscope was able to simultaneously measure the heart sounds and PPG signals with a high accuracy similar to that of professional physiological systems. In addition, within the developed automatic heart-sound-analysis algorithm based on a PPG signal reference, the proposed device proved to be highly accurate in automatic S1 and S2 signal identification.

For future development, the head structure of the proposed smart stethoscope will be further improved based on acoustic theory to maximize the resolution of sound measurement. Future iterations of the device will also be considered, such as allowing the smart stethoscope to be used over clothing, or to be used while walking, running, or sleeping.

## 5. Conclusions

In this paper, a finger-ring-shaped hybrid personal smart stethoscope with an automatic heart-sound-analysis function was presented. Different from common single head stethoscopes, the proposed smart stethoscope was implemented with an automatic heart-sound-analysis algorithm based on a PPG signal reference that was simultaneously measured with the heart sounds to automatically identify S1 and S2 heart sounds. To achieve this goal, the proposed smart stethoscope was developed with a finger-ring shape for personal one-hand usage, and was able to simultaneously measure the heart sounds and PPG signals through its novel design: one side was attached to the surface of the chest for heart-sound acquisition, and the other side was attached to the finger to measure PPG signals. The proposed smart stethoscope was manufactured as a prototype device to evaluate its performance in heart-sound and PPG-signal measurement, and to observe the accuracy of the developed algorithm in automatic identification of S1 and S2 heart sounds. Through an in vivo experiment with 20 subjects, the experimental results showed that the prototype device was highly accurate, with results similar to those of professional physiological acquisition systems. In addition, the experimental results showed the developed heart-sound-analysis function based on calculating the time interval between the start-point and PPG signal peaks could automatically identify S1 and S2 heart sounds accurately. Furthermore, with a 400 mAh rechargeable battery and a low-power, high-performance BLE SoC ARM processor, the power consumption of the prototype in operation status was 30 mAh, giving it a battery life of more than 10 h per charge. The manufactured prototype device demonstrated that the proposed finger-ring-shaped hybrid personal smart stethoscope can be used for self-monitoring of heart sounds anywhere and anytime.

## Figures and Tables

**Table 1:** Cross-correlation values for heart sounds and PPG signals measured by the prototype device and the MP160.

Signal Group	Heart Sounds		PPG Signals	
	Mean	Std.	Mean	Std.
Original	0.98	0.02	0.96	0.03

## Data Availability

The dataset supporting the conclusions of this article is not available due to privacy and ethical reasons.
